# Socioeconomic and Geographic Disparities in Anorectal and Urinary Procedures Following Radiotherapy for Prostate Cancer

**DOI:** 10.1002/cam4.71135

**Published:** 2025-08-05

**Authors:** Tenaw Tiruye, Braden Higgs, Michael O'Callaghan, Liesel M. FitzGerald, David Roder, Kerri Beckmann

**Affiliations:** ^1^ Cancer Epidemiology and Population Health Research Group Allied Health and Human Performance, University of South Australia Adelaide Australia; ^2^ School of Public Health, Debre Markos University Debre Markos Ethiopia; ^3^ Department of Radiation Oncology Royal Adelaide Hospital Adelaide Australia; ^4^ Flinders Medical Centre Bedford Park Australia; ^5^ South Australian Prostate Cancer Clinical Outcomes Collaborative Adelaide Australia; ^6^ Flinders Health and Medical Research Institute, Flinders University Adelaide Australia; ^7^ Discipline of Medicine University of Adelaide Adelaide Australia; ^8^ Menzies Institute for Medical Research, University of Tasmania Hobart Australia

**Keywords:** disparity, prostate cancer, radiotherapy, treatment outcome

## Abstract

**Background and Purpose:**

Evidence on how treatment outcomes vary by patient characteristics helps to inform clinical practice. In this study, we measured socioeconomic and geographic disparity in post‐radiotherapy procedures, as an indication of short‐term radiotherapy adverse effects, among men with prostate cancer.

**Materials and Methods:**

We studied 8344 South Australian diagnosed men with prostate cancer (2002–2020) who received external beam radiotherapy. The outcomes were anorectal and urinary procedures, identified using hospital admission procedure codes and Medicare Benefits Schedule item codes. Crude rates per 1000 person‐time were estimated at two years post‐radiotherapy. Socioeconomic and geographic disparities were identified through multivariable adjusted zero‐inflated Poisson regression.

**Results:**

Fifteen percent of men underwent at least one post‐radiotherapy procedure within two years. The rates of anorectal, urinary and overall (both anorectal and urinary) procedures were 18, 66 and 81 per 1000 person‐years, respectively. Men in the highest socioeconomic quintile had lower rates of overall (incidence rate ratio [IRR] 0.70, 95% CI: 0.61–0.81), anorectal (IRR 0.32, 95% CI: 0.20–0.52) and urinary (IRR 0.69, 95% CI: 0.56–0.86) procedures than men in the lowest socioeconomic quintile. Men from non‐metropolitan areas had higher rates of anorectal procedures (IRR 1.36, 95% CI: 1.05–1.77) than men from metropolitan areas, which was further compounded by low socioeconomic advantage. Receiving radiotherapy in more recent years was associated with lower rates of post‐radiotherapy procedures.

**Conclusion:**

Anorectal and urinary procedures following radiotherapy significantly vary across different population subgroups. Observed differences in procedure rates may suggest socioeconomic and geographic disparities in radiotherapy adverse effects for prostate cancer. This underscores the importance of follow‐up care for at‐risk population subgroups.

**Abbreviations**
ABS: Australian Bureau of Statistics
ACHI: Australian Classification of Health Interventions
ADT: androgen deprivation therapy
CI: Ccnfidence interval
IRR: incidence rate ratio
ISAAC: Integrated South Australian Activity Collection
MBS: Medicare Benefits Schedule
SA‐PCCOC: South Australian Prostate Cancer Clinical Outcomes Collaborative
SDoH: social determinants of health
SEIFA: Socio‐Economic Indices for Areas

## 1 Introduction

Prostate cancer is the most common cancer and the second leading cause of cancer‐related deaths in Australian males [1]. Radiotherapy continues to be a common treatment option for prostate cancer. However, several studies have shown that men who undergo radiotherapy may experience sexual dysfunction and bowel problems [2, 3, 4, 5] and are at increased risk of developing secondary malignancies [6, 7].

Advances in screening, treatment and imaging technologies have led to a 96% 5‐year survival rate among affected men [8]. However, not all men have equal access to these diagnostic and treatment modalities, and cancer outcomes vary [9, 10, 11]. Reducing inequalities and ensuring essential services are consistently and widely accessible to all populations has become an increasingly important focus of cancer control efforts, alongside improving survival and quality of life [12].

In Australia, higher socioeconomic disadvantage is generally associated with poorer survival [13, 14], lower PSA testing rates [14], more advanced cancer [13, 14, 15] and decreased quality of life [16]. Men living in rural and remote areas are significantly more likely to have lower rates of PSA testing [9], be diagnosed at a later age [10], have more clinically aggressive cancer [10, 15], have poorer survival outcomes [9] and take longer to commence active treatment [10]. Lack of access to diagnostic and treatment services may lead to later stage disease at diagnosis, deter or restrict patients from accessing or completing treatment, and lead to disparities in treatment outcomes among patients from rural settings and/or disadvantaged communities. Evidence also shows disparities in the quality of care, with urban men more likely to receive treatment from high‐volume surgeons [17] or at comprehensive care facilities [18]. Furthermore, a combination of structural and systemic barriers—including poor access to specialised cancer centres, transportation issues and long travel times, financial constraints and lower health literacy—faced by people from disadvantaged communities contributes to delays in diagnosis and treatment, as well as post‐treatment follow‐up care leading to worsening health conditions and a need for more intensive healthcare interventions [19, 20].

However, there is scarce evidence regarding disparities in adverse events that require hospitalization and/or medical intervention post prostate cancer treatment, including after radiotherapy. One reason for this lack of evidence is a paucity of appropriate data. To overcome this, we linked a prostate cancer clinical registry to state and national health registries (linkage completed in 2022) as data linkage using population‐based and disease‐specific registries has been shown to play an important and growing role in advancing prostate cancer research and care [21]. In this study of men with prostate cancer, we aimed to measure socioeconomic and geographic disparities in post‐radiotherapy anorectal and urinary procedures (as indicators of radiotherapy adverse effects).

## 2 Materials and Methods

### 2.1 Study Population

This population‐wide study included 8344 South Australian men diagnosed with prostate cancer between 2002 and 2020 who were treated with external beam radiotherapy. Men who received radiotherapy following their prostate cancer diagnosis were identified through treatment records within the South Australian Prostate Cancer Clinical Outcomes Collaborative registry (SA‐PCCOC), hospital admission procedure codes from the Integrated South Australian Activity Collection (ISAAC), and Medicare Benefits Schedule (MBS) item codes (Table S1). While our linked data did not include private hospital separations data from ISAAC, procedures performed in the private sector were captured through MBS. This means that procedures conducted in both public and private settings were extractable from our linked datasets. Men who had a radical prostatectomy within two years before or after the date of radiotherapy (*n* = 2014) were excluded to avoid additive effects of these two common active treatments for prostate cancer. Additionally, patients with a missing postcode (*n* = 958) were excluded as it was not possible to determine their socioeconomic (dis)advantage or rurality/remoteness without this information. Men who had androgen deprivation therapy (ADT) within 2 years of radiotherapy (*n* = 3180) were included, with adjustment for ADT in the final models.

### 2.2 Measurement and Variables

The primary outcomes of interest were anorectal, urinary and overall (both anorectal and urinary) procedures as proxy measures of radiotherapy adverse effects, with the focus being on short‐term outcomes (within two‐year post‐radiotherapy). Twenty‐two procedures (seven anorectal and 15 urinary) were preselected in consultation with a Radiation Oncology and a Urology consultant for inclusion in the analyses. These were extracted from ISAAC and MBS using the relevant Australian Classification of Health Interventions (ACHI) procedure codes and MBS item codes (Table S2).

Socioeconomic advantage was derived from the Australian Bureau of Statistics (ABS) Socio‐Economic Indices for Areas (SEIFA). SEIFA ranks areas in Australia according to relative socio‐economic advantage and disadvantage based on information from the five‐yearly Australian Census of Population and Housing. In our analyses, we have used the Index of Relative Socio‐economic Advantage and Disadvantage (IRSAD) version of SEIFA which provides a summary measure of the socio‐economic conditions of people and households at the postal area level, encompassing both advantage and disadvantage. A low IRSAD score indicates relative greater disadvantage (e.g., many households with low incomes or many people in unskilled occupations), while a high score suggests greater advantage (e.g., many households with high income or many people with high education levels) [22]. We applied the SEIFA‐IRSAD scores for the census year (2001, 2006, 2011 and 2016) closest to the patient's prostate cancer diagnosis date and categorised scores into quintiles from lowest socioeconomic advantage (greater disadvantage) to highest socioeconomic advantage (least disadvantaged). Place of residence was grouped as ‘Metropolitan/Greater Adelaide area (metropolitan) and rest of South Australia (non‐metropolitan residents) based on the ABS's Statistical Areas Level 3 (SA3‐2016) data for patients’ residential address at diagnosis [23].

Other potential covariates included age at diagnosis, comorbidity, year of treatment, and whether received ADT. Patient's age at diagnosis was categorised as < 60, 60–64, 65–69, 70–74 and ≥ 75 years. Pre‐existing comorbidity was measured using Rx‐Risk, a prescription‐based comorbidity index, which has previously been validated in our cohort [24, 25], with a look‐back period of 1 year prior to prostate cancer diagnosis. Rx‐Risk was grouped as 0, 1, 2 and ≥ 3, as previously suggested [24]. Year of treatment was grouped as 2002–2005, 2006–2010, 2011–2015 and 2016–2021.

### 2.3 Statistical Analyses

The frequencies and percentages of overall count of relevant procedures within 2‐years of radiotherapy were compared across strata within variables, and differences were assessed using chi‐square tests. The rates of adverse events per 1000 person‐years were calculated, with rates adjusted for variable follow‐up times and censoring due to death. Zero‐inflated Poisson regression models were applied to identify socioeconomic and geographic disparities in the rates of overall, anorectal and urinary procedures within two years following radiotherapy. Risk of overall, anorectal and urinary procedures were modeled separately. To further assess whether belonging to multiple disparity domains (e.g., rural men from least socioeconomic advantaged areas) increased the risk of post‐radiotherapy procedures, we used postestimation marginal probability prediction after logistic regression models and presented results graphically. Analyses were performed using Stata18 (StataCorp).

## 3 Results

A total of 8344 men were included in the analyses. The mean age at diagnosis was 70 years (standard deviation ±7.8). The majority resided in the metropolitan area (73%) and 22% were categorized as being in the lowest socioeconomic advantage quintile (Table 1).

**TABLE 1:** Patient characteristics (*N* = 8344).

Variables	Total		Had any procedure in 2 years				
			No		Yes		*p*
	No.	%	No.	%	No.	%	
Total	8344	100	7106	85.2	1238	14.8	
Age at diagnosis							
< 60	827	9.9	726	10.2	101	8.2	< 0.001
60–64	1091	13.1	954	13.4	137	11.1	
65–69	1682	20.2	1465	20.6	217	17.5	
70–74	2097	25.1	1770	24.9	327	26.4	
75+	2647	31.7	2191	30.8	456	36.8	
Mean ± SD	70.2 ± 7.8		70.0 ± 7.9		71.1 ± 7.4		
Socioeconomic advantage							
Lowest (most disadvantage)	1791	21.5	1484	20.9	307	24.8	< 0.001
Low	1600	19.2	1345	18.9	255	20.6	
Average	1764	21.1	1514	21.3	250	20.2	
High	1739	20.8	1478	20.8	261	21.1	
Highest (least disadvantage)	1450	17.4	1285	18.1	165	13.3	
Place of residence							
Metropolitan	6120	73.3	5169	72.7	951	76.8	0.003
Non‐metropolitan	2224	26.7	1937	27.3	287	23.2	
Year of treatment							
2002–2005	1087	13.0	915	12.9	172	13.9	0.039
2006–2010	2377	28.5	1991	28.0	386	31.2	
2011–2015	2107	25.3	1803	25.4	304	24.6	
2016–2021	2773	33.2	2397	33.7	376	30.4	
Rx‐Risk comorbidity index							
0	1515	18.2	1330	18.7	185	14.9	0.009
1	1388	16.6	1188	16.7	200	16.2	
2	1307	15.7	1101	15.5	206	16.6	
3+	4134	49.5	3487	49.1	647	52.3	
Received ADT							
No	5164	61.9	4387	61.7	777	62.8	0.493
Yes	3180	38.1	2719	38.3	461	37.2	

Abbreviations: ADT, androgen deprivation therapy; SD, standard deviation.

Table 2 summarises rates of procedures. Overall, the rate of any of the selected procedures two years post‐radiotherapy was 81 per 1000 person‐years, with rates of anorectal and urinary procedures being 18 and 66 per 1000 person‐years, respectively. The most common anorectal procedures were for anorectal stricture (9/1000 person‐year) and proctitis (6/1000 person‐year), while the most common urinary procedures were cystoscopy (56/1000 person‐year) and urethroscopy (46/1000 person‐year).

**TABLE 2:** Rates per 1000 person‐years of post‐radiotherapy procedures (*N* = 8344).

Procedures	*N*	Rate	Rate 95% CI	
Anorectal				
Anorectal stricture	143	9.4	8.0	11.1
Anorectal fistula/fissure	11	0.7	0.4	1.3
Anal/faecal incontinence	< 6	—	—	—
Proctitis	98	6.4	5.3	7.8
Proctetectomy	10	0.7	0.4	1.2
Resection of rectum	23	1.5	1.0	2.3
Anorectal abscess/thrombus	9	0.6	0.3	1.1
Urinary				
Overactive bladder	< 6	—	—	—
Bladder catheterisation	204	13.4	11.7	15.4
Cystoscopy	856	56.2	52.6	60.1
Bladder repair/excision	8	0.5	0.3	1.1
Cystostomy	34	2.2	1.6	3.1
Cystectomy	< 6	—	—	—
Vesical fistula	< 6	—	—	—
Incontinence	< 6	—	—	—
Urethral stricture	54	3.6	2.7	4.6
Urethroscopy	704	46.2	42.9	49.8
Urethroplasty	< 6	—	—	—
Urethrotomy	107	7.0	5.8	8.5
Urethrectomy	< 6	—	—	—
Urethral fistula/rupture	< 6	—	—	—
Artificial sphincter	< 6	—	—	—
Any anorectal procedure	277	18.2	16.2	20.5
Any urinary procedure	1007	66.1	62.2	70.3
Overall procedure	1238	81.3	76.9	85.9

*Note:* Procedures with < 6 observations in a cell are not reported due to data use agreement.

In the multivariable adjusted models (adjusted for age, comorbidity, year of treatment and ADT), men residing in areas of higher compared with lower socioeconomic advantage had 30% lower rates of overall procedures (highest vs. lowest: incidence rate ratio [IRR] 0.70, 95% CI 0.61–0.81). Likewise, men in the highest socioeconomic advantage quintile had a 68% lower rate of anorectal (IRR 0.32, 95% CI 0.20–0.52) and a 31% lower rate of urinary procedures (IRR 0.69, 95% CI 0.56–0.86) than men in the lowest socioeconomic advantage quintile. Regarding place of residence, non‐metropolitan South Australian men had a 36% higher rate of anorectal procedures (IRR 1.36, 95% CI: 1.05–1.77) than men living in the capital city (Greater Adelaide area). However, there was no statistically significant difference in the rate of overall and urinary procedures by place of residence (Table 3).

**TABLE 3:** Multivariable zero inflated Poisson regression outputs, procedures within 2‐years of radiotherapy.

Variables	Overall procedure				Anorectal procedure				Urinary procedure			
	IRR	95% CI		*p*	IRR	95% CI		*p*	IRR	95% CI		*p*
Socioeconomic advantage												
Lowest (most disadvantage)	Ref				Ref							
Low	0.91	0.80	1.03	0.120	0.90	0.64	1.25	0.524	0.91	0.74	1.11	0.352
Average	**0.88**	**0.78**	**1.00**	**0.049**	0.87	0.72	1.04	0.132	0.92	0.75	1.13	0.437
High	**0.83**	**0.73**	**0.94**	**0.004**	**0.69**	**0.48**	**0.98**	**0.040**	0.83	0.68	1.02	0.076
Highest (least disadvantage)	**0.70**	**0.61**	**0.81**	**0.000**	**0.32**	**0.20**	**0.52**	**0.000**	**0.69**	**0.56**	**0.86**	**0.001**
Place of residence												
Metropolitan	Ref				Ref							
Non‐metropolitan	1.01	0.78	1.30	0.959	**1.36**	**1.05**	**1.77**	**0.022**	0.92	0.78	1.07	0.271
Age at diagnosis												
< 60	Ref				Ref							
60–64	1.02	0.77	1.34	0.914	1.03	0.80	1.33	0.817	1.03	0.76	1.40	0.832
65–69	1.02	0.88	1.19	0.765	1.04	0.79	1.36	0.784	1.12	0.85	1.48	0.414
70–74	1.22	0.96	1.57	0.108	**1.28**	**1.00**	**1.63**	**0.046**	1.22	0.94	1.60	0.140
75+	**1.36**	**1.07**	**1.74**	**0.012**	**1.42**	**1.12**	**1.80**	**0.004**	**1.50**	**1.15**	**1.94**	**0.003**
Year of treatment												
2002–2005	Ref				Ref							
2006–2010	0.95	0.83	1.09	0.459	0.89	0.78	1.02	0.098	0.94	0.76	1.16	0.553
2011–2015	0.87	0.71	1.07	0.189	**0.59**	**0.39**	**0.89**	**0.012**	0.92	0.73	1.14	0.427
2016–2021	**0.81**	**0.66**	**0.99**	**0.039**	**0.57**	**0.39**	**0.85**	**0.005**	**0.76**	**0.62**	**0.94**	**0.010**
Rx‐Risk comorbidity index												
0	Ref				Ref							
1	1.18	0.95	1.47	0.132	1.34	0.86	2.09	0.193	1.07	0.85	1.35	0.548
2	**1.20**	**1.04**	**1.39**	**0.013**	1.43	0.91	2.25	0.119	1.21	0.96	1.53	0.103
3+	**1.27**	**1.06**	**1.53**	**0.011**	**1.62**	**1.11**	**2.38**	**0.013**	**1.24**	**1.04**	**1.48**	**0.016**
Received ADT												
No	Ref				Ref							
Yes	0.93	0.84	1.03	0.183	0.97	0.86	1.11	0.677	0.90	0.78	1.03	0.125

*Note:* Statistically significant associations are highlighted bold. Interpretation of IRR: IRR of 1 (or 95% CI including 1) indicates no significant difference in the risk of post‐radiotherapy procedures between groups (e.g., ‘low’ vs. ‘lowest’ socioeconomic advantage). IRR > 1 (and 95% CI > 1) signifies a higher risk in the group of interest compared to the reference group (e.g., non‐metropolitan patients have a 36% higher risk than metropolitan patients). IRR < 1 (and 95% CI < 1) indicates a lower risk in the group of interest than the reference group (e.g., patients with highest socioeconomic advantage have a 30% lower risk than patients with most disadvantaged).

Abbreviations: ADT, androgen deprivation therapy; CI, confidence interval; IRR, adjusted incidence rate ratio; Ref, reference category.

**FIGURE 1:**
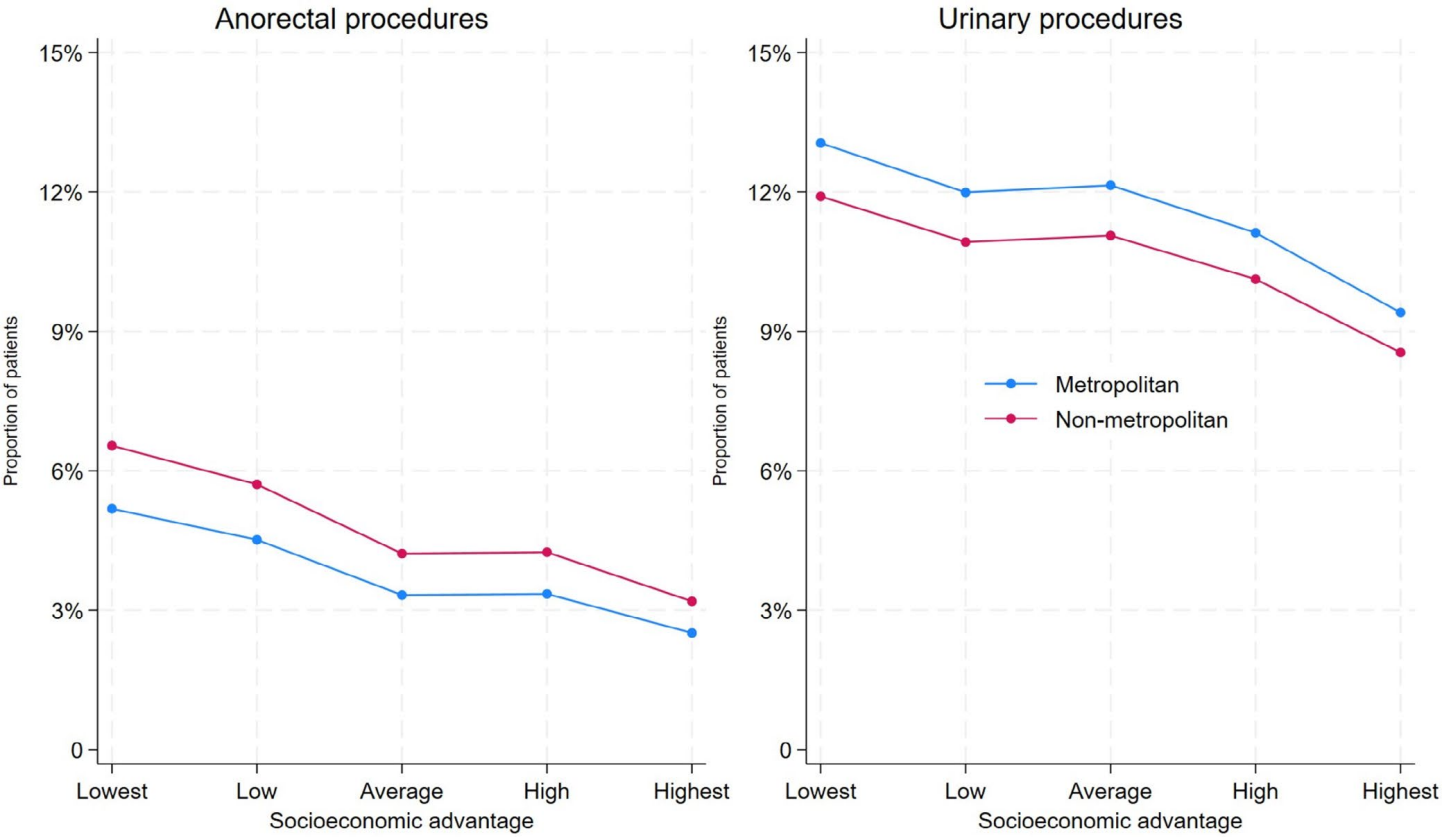
Predicted probability of post‐radiotherapy anorectal and urinary procedures, stratified by socioeconomic advantage and place of residence.

Further investigation assessing both regional and socioeconomical disadvantage indicated that men residing in the least socio‐economically advantaged non‐metropolitan areas had a higher probability of undergoing anorectal procedures than their urban counterparts living in the highest socioeconomic areas (6.5% vs. 2.5% probability). Additionally, regardless of socioeconomic status, metropolitan residents have lower anorectal procedure rates. However, in terms of urinary procedures, men residing in metropolitan areas seem to have higher urinary procedure rates, irrespective of socioeconomic status (Figure 1, Table S3).

Other factors associated with higher risk of both anorectal and urinary procedures were older age (75+ vs. < 60 years) and having a high comorbidity burden (Rx‐Risk score 3+ vs. 0). In contrast, men who were treated from 2016 to 2021 had 43% lower risk of anorectal procedures (IRR 0.57, 95% CI: 0.39–0.85) and 24% lower risk of urinary procedures (IRR 0.76, 95% CI: 0.62–0.94) compared with patients who were treated from 2002 to 2005. The use of ADT with radiotherapy was not associated with risk of subsequent procedures (Table 3).

## 4 Discussion

This study investigated the rates of anorectal, urinary and overall (both anorectal and urinary) procedures within 2 years of undergoing radiotherapy for prostate cancer, as a proxy indicator of short‐term radiotherapy adverse effects. Our findings indicate significant disparities in the occurrence of these procedures, with individuals from the least socioeconomic advantage and nonmetropolitan areas being disproportionately affected.

Our findings align with reported disparities in prostate cancer outcomes across various measures in Australia, including low PSA testing rates [9, 14], advanced stage diagnoses [10, 13, 14, 15], poorer survival [9, 13, 14] and decreased quality of life [16] among men in rural/remote and/or socioeconomically disadvantaged communities. Disparity in prostate cancer outcomes is not specific to Australia only. Reviews of international patterns consistently show that men from socioeconomically disadvantaged and rural areas experience poorer prostate cancer outcomes across the entire prostate cancer prevention and control continuum (from screening, diagnosis, treatment, through to survivorship care), including lower PSA testing, higher advanced disease at diagnosis, and reduced survival [26, 27, 28, 29, 30]. Collectively, these findings reinforce the universality of structural and systemic challenges in achieving health equity both within Australia and globally and the need to work cooperatively toward achieving health equity in prostate cancer control and management.

The observed disparities can likely be attributed to a complex interplay of underlying factors, including lifestyle and behavioral factors and access to healthcare. Studies show that low socioeconomic status could have an additive role in the relationship between behavioral risk factors and adverse health outcomes [31]. Likewise, compared to people living in cities, those in rural Australia are more likely to engage in insufficient exercise, inadequate fruit intake relative to national guidelines, and risky alcohol consumption [32]. Lifestyle and behavioral risks are well‐established as being associated with poorer cancer outcomes [33, 34, 35, 36]. However, it is crucial to view these risks through the lens of the influence systemic and environmental factors have on shaping health behaviors. For instance, limited access to affordable and healthy food options, the absence of safe and accessible spaces for physical activity, and insufficient access to preventive services are not merely individual choices but rather consequences of broader structural inequities. A study from the US reported that disadvantaged neighborhoods experienced food insecurity and are often also characterized by a disproportionately high prevalence of fast‐food businesses, liquor stores, low‐income housing units and a lack of safe and affordable physical exercise options [19]. Such environments, compounded by geographic isolation and economic constraints, foster a cycle of lifestyle habits that fundamentally limit an individual's ability to engage in health‐promoting behaviors, make individuals more susceptible to complications and impact their disease recovery.

Although the Australian healthcare system is universal, patients from remote and regional areas face significant barriers due to distance and limited access to specialised care. This is particularly notable in South Australia, where approximately 78% of the population resides in the capital city [37], and hence most healthcare services are concentrated there. Further investigation is needed to determine whether the observed variations in our study reflect disparities in prostate cancer care and service provision. A previous study also indicated that men in rural Australia are more likely to have their prostate cancer diagnosed incidentally during other health procedures, rather than through proactive screening or monitoring [11]. This suggests that rural men are more likely to be diagnosed with late‐stage or advanced disease, which may result in poor treatment outcomes and a worse prognosis.

A previous Australian study of 1526 patients enrolled in the Prostate Cancer Outcomes Registry‐Tasmania indicated that men from outer regional/remote areas were significantly more likely to also be residing in a low socioeconomic area [10]. In this regard, our study showed that men who belonged to multiple disparity domains (i.e., men from low socioeconomic non‐metropolitan areas) had even higher rates of anorectal procedures, demonstrating a synergistic effect of these disparity domains on poorer treatment outcomes. This could also reflect a delayed presentation for care. Such delays in receiving an early diagnosis can lead to a more advanced disease stage, potentially necessitating higher doses and larger radiotherapy volumes, thus increasing toxicity risk. After initial treatment, persistent disparities in timely follow‐up care can arise from a combination of factors: limited availability of specialized cancer centers, poor access to transportation, long travel times, insufficient information to understand treatment side effects, miscommunication between providers and patients, or inadequate patient navigation services. Since all prostate cancer radiotherapy services in South Australia are concentrated in metropolitan Adelaide, and all men are likely treated by the same experienced centers, the observed disparities are unlikely to be due to physician‐ or center‐related factors; rather, there is a possibility that the observed differences are highly attributable to follow‐up care after radiotherapy. Interruptions in follow‐up care can worsen post‐treatment complications that might have been detected and addressed sooner, thereby increasing the need for additional healthcare interventions. The higher procedure rates seen in disadvantaged groups, therefore, could indicate a combination of advanced disease severity upon diagnosis and the exacerbation of minor post‐treatment complications.

In addition, this study highlights the impact of social determinants of health (SDoH) through which socioeconomic disadvantage and geographic isolation could drive disparities in health outcomes [19, 38]. SDoH posits that an individual's health and quality of life are significantly shaped by where they are born, live, work and age [19, 20]. Factors such as limited healthcare access, issues with transportation, financial constraints, lower health literacy, unemployment and systemic discrimination can contribute to unequal access to cancer screening, early diagnosis, and treatment, and survivorship care [19]. The combined impact of socioeconomic disadvantage and geographic isolation can limit access to preventive care, timely diagnosis and adequate and consistent follow‐up care [19]. Such systemic and structural deficiencies can lead to delays in seeking care, leading to disease progression, worse health outcomes and the eventual need for more complex and invasive procedures, thereby exacerbating health disparities [39]. Global literature also points to how implicit bias among healthcare providers and systemic biases within healthcare systems can contribute to unequal treatment and outcomes, particularly for racial and ethnic minorities who are often overrepresented in socioeconomically disadvantaged and geographically isolated areas [40, 41]. Therefore, the observed disparities in anorectal and urinary procedures after radiotherapy likely represent downstream effects of the broader SDoH.

While this study primarily focused on socioeconomic and geographic disparities, the observed differences could also reflect other population level differences such as being an indigenous Australian or from a culturally and linguistically diverse (CALD) background. Previous studies showed that indigenous Australians are more likely to be diagnosed with advanced cancers [42] and have a higher probability of cancer death [14, 42, 43] than other Australians, with one study showing that Aboriginal men are 49% more likely to die from prostate cancer [43]. Given that Australian indigenous communities are overrepresented in socioeconomically disadvantaged and regional/remote areas [44], these intersecting factors may compound disparities in post‐radiotherapy outcomes. A recent Australian study also showed that people from CALD backgrounds have more advanced prostate cancer at diagnosis [45]. Future research could explore whether the intersectionality of disadvantage (e.g., socioeconomic status, geography and other disparity domains in the population) and socioeconomic and systemic factors could further increase the risk of poorer cancer outcomes.

It is likely that the reported disparities in our study are underestimated. First, men from rural or remote areas, as well as those with lower socioeconomic status, are less likely to seek timely medical attention for post‐treatment adverse events due to financial constraints, long travel distances and transportation costs. For instance, previous research indicates significant inequalities in access to prostate‐specific membrane antigen positron emission tomography (PSMA PET) [46] and in receiving radiotherapy [47], with lower rates observed among regional/remote communities. Urban men generally have better access to follow‐up services and/or investigative procedures, such as cystoscopy (the most common urinary procedure in this study), which may lead to an increased reporting of urinary procedures.

It is encouraging to find that men treated in more recent years (2016–2021) exhibited lower rates of post‐radiotherapy procedures compared to those treated in earlier years (2002–2005). The decline in procedure rates over time may reflect recent advancements in radiotherapy delivery, such as the use of Intensity‐Modulated Radiation Therapy (IMRT) and Image‐Guided Radiation Therapy (IGRT) [48, 49]. Pre‐, during and post‐radiotherapy supportive care—such as exercise [50] and multidisciplinary rehabilitation interventions [51, 52]—has been shown to enhance post‐radiotherapy functional outcomes. Greater attention to improving patients' quality of life through offering such interventions may have also contributed to the positive trends observed in post‐radiotherapy outcomes. Other possible reasons include the contributions of prostate cancer nurse specialists in enhancing the focus on quality of life and functional outcomes, as well as the use of rectal spacers during radiotherapy to reduce radiation exposure in neighboring tissues.

Our findings suggest that patients residing in non‐metropolitan areas or disadvantaged communities may benefit from more intensive follow‐up protocols and management of potential complications. Additionally, healthcare providers should be aware of these risk profiles and develop appropriate follow‐up care plans. A multidisciplinary approach that encompasses comprehensive patient care, including early detection, timely intervention and effective management of potential adverse effects, is essential. Improving the availability of specialized care in disadvantaged areas and implementing targeted interventions, such as addressing structural and environmental factors influencing lifestyle and behavior, may help reduce disparities. The higher rates of post‐radiotherapy procedures observed in disadvantaged populations may also reflect delayed care and worsened health outcomes, highlighting a critical need for proactive measures such as early detection and timely preventive measures to avert complications from worsening. Integrating these prevention strategies, alongside more targeted interventions to address barriers to timely care, would help mitigate future health complications and reduce the need for more invasive procedures down the line.

This study has the following potential limitations. First, the study is observational in nature, which limits the ability to draw causal inferences. Second, the reported procedures may not be solely attributable to radiotherapy and can only serve as proxy measures for adverse events rather than indicating actual complications from radiotherapy. Due to the lack of diagnosis codes from hospital records, we could not determine the reasons for which procedures were undertaken. Hence, the observed disparities may reflect variations in the general frequency of genitourinary and gastrointestinal health issues within these patient groups rather than true radiotherapy‐related complications. Third, although we have adjusted for comorbidity variables using Rx‐Risk, the possibility of residual confounding remains. Hence, conditions not captured by Rx‐Risk may contribute to the observed disparities. Fourth, we were unable to make comparisons in terms of dose, fractionation or radiotherapy modality and to pinpoint the precise reasons for the observed disparities in radiotherapy toxicities across different patient groups.

Despite the above limitations, the strengths of our study are its large, comprehensive, population‐wide scope and its use of real‐world data to capture procedures undertaken in both public hospitals (through ACHI) and private practice (through linked MBS data). This study also demonstrates that improved access to linked health data enables a comprehensive analysis of treatment outcomes at the population level, which may facilitate the development of targeted interventions to reduce cancer disparities among different population subgroups.

## 5 Conclusion

The differences in anorectal and urinary procedure rates observed in our study are likely to indicate socioeconomic and regional disparities in treatment‐related adverse events following radiotherapy for prostate cancer. These findings underscore the need for comprehensive follow‐up care plans to effectively prevent and/or manage potential adverse events following radiotherapy for prostate cancer, especially for subgroups at greatest risk.

## Author Contributions

**Tenaw Tiruye:** conceptualization (equal), data curation (equal), formal analysis (equal), investigation (equal), methodology (equal), project administration (equal), validation (equal), writing – original draft (equal), writing – review and editing (equal). **Braden Higgs:** conceptualization (equal), methodology (equal), writing – review and editing (equal). **Michael O'Callaghan:** methodology (equal), writing – review and editing (equal). **Liesel M. FitzGerald:** methodology (equal), writing – review and editing (equal). **David Roder:** data curation (equal), funding acquisition (equal), project administration (equal), supervision (equal), writing – review and editing (equal). **Kerri Beckmann:** conceptualization (equal), data curation (equal), formal analysis (equal), investigation (equal), methodology (equal), project administration (equal), writing – review and editing (equal).

## Ethics Statement

Ethics approval was obtained from the South Australia Department for Health and Wellbeing Human Research Ethics Committee (HREC/20/SAH/58) and Australian Institute of Health and Welfare Ethics Committee (EO2020/5/1202).

## Consent

The authors have nothing to report.

## Conflicts of Interest

The authors declare no conflicts of interest.

## Supporting information


**Table S1:** Procedures codes used to identify prostate cancer radiotherapy.
**Table S2:** Australian Classification of Health Interventions (ACHI) procedure codes and Medicare Benefits Schedule (MBS) item codes.
**Table S3:** Postestimation marginal prediction of the probability of post‐radiotherapy anorectal and urinary procedures, stratified across socioeconomic advantage and place of residence.

## Data Availability

The linked datasets that support the findings of this study are stored in the Secure Unified Research Environment (SURE) system, where restrictions apply regarding data access, and so are not publicly available. However, data are available from the authors upon reasonable request and with permission from the data custodians.
