# Supplementary material for: Socioeconomic and Geographic Disparities in Anorectal and Urinary Procedures Following Radiotherapy for Prostate Cancer
Source: Cancer Med. 2025 Aug 5;14(15):e71135. doi: 10.1002/cam4.71135 (PMC12324824; doi:10.1002/cam4.71135)
Supplement: Supplementary file 1 — Table S1: Procedures codes used to identify prostate cancer radiotherapy. Table S2: Australian Classification of Health Interventions (ACHI) procedure codes and Medicare Benefits Schedule (MBS) item codes. Table S3: Postestimation marginal prediction of the probability of post‐radiotherapy anorectal and urinary procedures, stratified across socioeconomic advantage and place of residence. [file CAM4-14-e71135-s001.docx]

Supplementary Table 1: Procedures codes used to identify prostate cancer radiotherapy

| SA-PCCOC | Hospital admission procedure codes | MBS item codes |
| --- | --- | --- |
| If stated so in the registry | 1522400 | 15233 |
|  | 1523900 | 15248 |
|  | 1526900 | 15263 |
|  | 1525400 | 15275 |
|  | 1560000 | 15218 |
|  | 1560001 | 15513 |
|  | 1520300 |  |
|  | 1520400 |  |
|  | 1520700 |  |
|  | 1520800 |  |

SA-PCCOC, South Australian Prostate Cancer Clinical Outcomes Collaborative registry, MBS, Medical Benefits Schedule.

Supplementary Table 2: Australian Classification of Health Interventions (ACHI) procedure codes and Medicare Benefits Schedule (MBS) item codes

| Procedure | | | ACHI procedure/MBS item codes |
| --- | --- | --- | --- |
| Anorectal | | Anorectal stricture | 32094, 32234, 32023, 32114, 32115, 30479, 32123, 32153 |
|  |  | Anorectal fistula/fissure | 32156, 32159, 32162, 32165, 32166, 90315, 32150, 90342 |
|  |  | Anal/faecal incontinence | 32126, 32129, 32220, 32221, 32203, 32206, 32210, 32213, 32214, 32215, 32216, 32217, 32218, 32237 |
|  |  | Proctitis | 32227, 32212 |
|  |  | Proctetectomy | 32047, 32039, 32042, 32045, 32060 |
|  |  | Resection of rectum | 32024, 32025, 32026, 32028, 32046, 92208 |
|  |  | Anorectal abscess/thrombus | 32147, 32174, 32175, 90338 |
| Urinary | Bladder | Overactive bladder | 36664, 36665, 36666, 36667, 36668, 36671, 36672, 36673, 90359 |
|  |  | Bladder catheterisation | 36800 |
|  |  | Cystoscopy | 36811, 36812, 36818, 36821, 36822, 36823, 36824, 36827, 36830, 36836, 36842, 36848, 36851, 36854 |
|  |  | Bladder repair/excision | 37000, 37004, 37014, 37020, 37050, 37053, 90360, 90363 |
|  |  | Cystostomy | 37008, 37011, 92121, 92102, 37026 |
|  |  | Cystectomy | 37016, 37018, 37019, 37021 |
|  |  | Vesical fistula | 37023, 37038 |
|  |  | Incontinence * | 37039, 37040, 37041, 37042, 37043, 37044, 37045, 37048, 37338, 37339, 37341, 18375, 18379 |
|  | Urethra | Urethral stricture | 37300, 37303, 90371, 90364 |
|  |  | Urethroscopy | 37318, 36811, 36812, 92120 |
|  |  | Urethroplasty | 37342, 37343, 37345, 37348, 37351, 37372 |
|  |  | Urethrotomy | 37321, 37324, 37327, 37354 |
|  |  | Urethrectomy | 37330 |
|  |  | Urethral fistula/rupture | 37336, 37306, 37309 |
|  |  | Artificial sphincter | 37375, 37381, 37384, 37387, 37388, 37390 |

** Some procedures in urethra are included here as some codes are not mutually exclusive*

Supplementary Table 3: Postestimation marginal prediction of the probability of post-radiotherapy anorectal and urinary procedures, stratified across socioeconomic advantage and place of residence

| Place of residence | Socioeconomic  advantage | Overall | | | Anorectal procedure | | | Urinary  procedure | | |
| --- | --- | --- | --- | --- | --- | --- | --- | --- | --- | --- |
|  |  | % | 95% CI | | % | 95% CI | | % | 95% CI | |
| Metropolitan (Greater Adelaide area) | Lowest | 17.8 | 16.0 | 19.6 | 5.2 | 4.2 | 6.2 | 13.1 | 11.5 | 14.6 |
|  | Low | 16.5 | 14.6 | 18.3 | 4.5 | 3.5 | 5.5 | 12.0 | 10.4 | 13.6 |
|  | Average | 15.3 | 13.4 | 17.1 | 3.3 | 2.5 | 4.2 | 12.1 | 10.5 | 13.8 |
|  | High | 15.9 | 14.0 | 17.7 | 3.4 | 2.5 | 4.2 | 11.1 | 9.7 | 12.6 |
|  | Highest | 12.7 | 10.9 | 14.5 | 2.5 | 1.8 | 3.2 | 9.4 | 8.0 | 10.8 |
| Non-metropolitan (rest of South Australia) | Lowest | 17.1 | 14.5 | 19.8 | 6.5 | 4.6 | 8.5 | 11.9 | 9.7 | 14.1 |
|  | Low | 15.9 | 13.5 | 18.2 | 5.7 | 4.1 | 7.3 | 10.9 | 9.0 | 12.8 |
|  | Average | 14.7 | 12.7 | 16.6 | 4.2 | 3.1 | 5.3 | 11.1 | 9.4 | 12.8 |
|  | High | 15.3 | 13.2 | 17.4 | 4.2 | 3.2 | 5.3 | 10.1 | 8.5 | 11.7 |
|  | Highest | 12.2 | 10.3 | 14.1 | 3.2 | 2.3 | 4.1 | 8.5 | 7.1 | 10.0 |
